# Interaction Insights: A new category for a growing journal

**DOI:** 10.1002/pei3.10033

**Published:** 2020-12-28

**Authors:** Wayne Dawson

**Affiliations:** ^1^ Department of Biosciences Durham University South Road, Durham DH1 3LE United Kingdom

Welcome to the third issue of *Plant‐Environment Interactions*. For this issue, I would like to take the opportunity to introduce you to a new category of article for our journal: *Interaction Insights*.

The word ‘insight’ can mean to truly apprehend the nature of an object or phenomenon, or the ability to see into inner character or underlying truth. As a journal, we recognise that sometimes, research findings do not necessarily fit the category of a full research article or review but may describe or reveal interesting information about interactions between plants and their environment. These findings deserve a venue for publication, even though they may be more descriptive and less hypothesis‐driven, because they will still give us an insight into how plants function within their environment. We are providing this venue by launching the category ‘*Interaction Insights*’ for more descriptive, discovery‐ or exploration‐based findings.

*Interaction Insights* will still be grounded in our philosophy of publishing sound science that adds to our understanding of plant‐environment interactions, and so will need to go beyond simple confirmatory accounts. For example, an ecologist may have analysed the microbial community associating with the roots of a particular species growing under certain environmental conditions, and they may find unusual plant‐microbe associations. Another example: a crop scientist might identify a fungal or viral pathogen on a particular crop, which has not been previously reported. A molecular plant scientist might discover changes in gene expression in plants in response to a particular environmental cue. A researcher studying plant physiology might describe physiological adaptations of an understudied plant to environmental stress. All these examples of research findings could certainly be published as *Interaction Insights*, because they are unusual or undescribed accounts of plant‐environment interactions.

There is currently no set format: short and concise is preferable, but as an online‐only journal, you should structure your *Insights* submission as you see fit. If a submission fits the category definition, we can then work with you to modify the manuscript as required. *Interaction Insights* will be subject to the same standards of peer review and data accessibility as our other categories.

We are keen as a journal to continue acting as an author‐friendly venue for publication of sound research in the plant and environmental sciences, and we hope that the new *Interaction Insights* category will help authors to broaden the range of research findings that they are able to publish in our field.

